# Sleep problems in pain patients entering tertiary pain care: the role of pain-related anxiety, medication use, self-reported diseases, and sleep disorders

**DOI:** 10.1097/j.pain.0000000000002497

**Published:** 2021-09-23

**Authors:** Teemu Miettinen, Jaana Sverloff, Olli-Pekka Lappalainen, Steven J. Linton, Kirsi Sipilä, Eija Kalso

**Affiliations:** a Department of Anaesthesiology, Intensive Care and Pain Medicine, University of Helsinki, Helsinki University Hospital, Helsinki, Finland; b Research Unit of Oral Health Sciences, University of Oulu, Oulu, Finland; c Department of Oral and Maxillofacial Diseases, University of Helsinki, Helsinki University Hospital, Helsinki, Finland; d Department of Law, Psychology and Social Work, Centre for Health and Medical Psychology, Örebro University, Örebro, Sweden; e Research Unit of Oral Health Sciences, University of Oulu, Oulu, Finland; f Oral and Maxillofacial Department, Medical Research Centre Oulu, Oulu University Hospital, Oulu, Finland; g Department of Pharmacology and SleepWell Research Programme, Faculty of Medicine, University of Helsinki, Helsinki, Finland

**Keywords:** Sleep disorder, Pain-related anxiety, Sleep medications, Comorbidities, Chronic pain

## Abstract

Chronic pain and sleep problems frequently co-occur. Pain itself disturbs sleep, but other factors may also contribute to sleep problems in pain patients. This cross-sectional study of 473 patients (69.9% female, mean age 47 years) entering tertiary pain management compared normally sleeping pain patients with those having recurring sleep problems to determine the relationship between pain and sleep. Groups were compared for pain and pain aetiology, pain-related anxiety, childhood adversities, use of sleep and pain medications, self-reported diseases, and sleep disorders. Furthermore, the association of pain-related anxiety (cognitive anxiety, escape/avoidance, fear, and physiological anxiety) with more disturbing sleep problems was investigated in the whole cohort. The main results were that those with sleep problems more often reported multiple health conditions than those sleeping normally (depression 31.6% vs 5.0%; angina pectoris 6.5% vs 0.0%; asthma 19.6% vs 1.7%; low back problems 55.1% vs 23.3%; joint disease other than rheumatoid arthritis 32.3% vs 18.3%). Accumulations of 5 or more childhood adversities were more often present in those with sleep problems. Restless legs symptoms were more common in those with sleep problems than those sleeping normally (33.2% vs 11.7%). Patients having sleep problems reported more use of sleep and pain medications than those sleeping normally. Findings about pain-related anxiety suggest physiological reactions as significant factors for increased sleep disturbances. These factors need to be addressed in the management of the comorbidity of pain and sleep problems, and research to understand mechanisms in these is sorely needed.

## Abstract 2

Supplemental Digital Content is Available in the Text.

Physiological anxiety reactions and a number of health conditions appeared as important factors differentiating chronic pain patients sleeping normally and with recurring sleep problems.

## 1. Introduction

Sleep problems are common among patients with chronic pain. They show clear associations with health-related quality of life (HRQoL) in pain patients,^54^ and resolving sleep problems may also alleviate pain.^16^

Pain is an obvious factor in sleep problems. Research has shown more intense and more widespread pain to be associated with greater sleep problems.^20,24,32^ However, other factors are also likely to contribute to sleep problems in pain patients. In chronic pain, anxiety reactions to pain are common. Worrying hinders falling or staying asleep,^43^ and ruminating thoughts about pain are found to be associated with greater sleep problems.^11^ Pain-related anxiety may also take other forms, such as fear and physiological anxiety reactions (eg, increased heart rate and blood flow).^34^ Physiological reactions especially may impair sleep,^9^ but there is little research about this in the context of pain. Adverse historical life experiences, more common among pain patients than in the general population,^27,30,53^ may increase sensitivity to stress,^22^ predisposing to greater sleep problems.^13^ Depression is also highly prevalent in chronic pain patients.^42^ Vicious circles may form as pain, anxiety, depression, and sleep problems reinforce each other.^3,18,20^

Somatic conditions,^28^ which may have triggered chronic pain development, as well as certain medications,^5^ may contribute to sleep problems, but research on this in pain patients is also scarce. Also, sleep disorders such as restless legs syndrome (RLS) and obstructive sleep apnoea (OSA) are more prevalent in pain patients than in the general population,^33^ but their prevalence among pain patients in tertiary care is less well known.

Sleep problems in pain patients are thus likely to be multifactorial, but studies broadly analysing these factors are lacking. The first aim of this study was to investigate how patients with sleep problems differ from normally sleeping pain patients regarding a diverse set of factors known or suspected to influence the development of poor sleep and, in turn, in some cases to be exacerbated by poor sleep: pain and pain aetiology (expecting more intense, more widespread, and longer-lasting pain to associate with poorer sleep^20,24,32^), components of pain-related anxiety (expecting cognitive anxiety to associate with poorer sleep^11^), childhood adversities (expecting more adversities to associate with poorer sleep^23^), use of sleep and pain medications, self-reported diseases (expecting depression and back pain problems to associate with poorer sleep),^2,18^ and the sleep disorders RLS and OSA (expecting both to associate with poorer sleep^33^). As a second aim, to further evaluate the role of pain-related anxiety components in disordered sleeping, we examined these components in the whole study cohort for their importance in more disturbing sleep problems. The information acquired was used to clarify the complexity of sleep problems and to help better target sleep interventions in patients with chronic pain.

## 2. Methods

### 2.1. Study design

**Figure 1.**
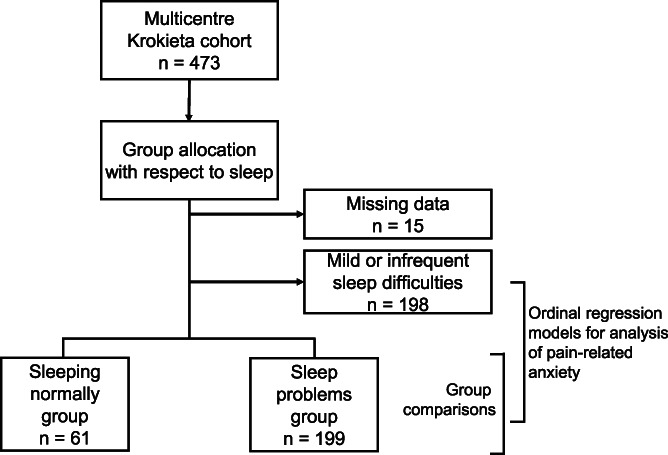
Data collection and analyses.

This was a cross-sectional study with a cohort comprising 473 patients entering tertiary pain management and participating in the multicentre (3 multidisciplinary pain clinics and 3 facial pain clinics) KROKIETA study. The cohort comprises chronic pain patients with mixed pain aetiologies, such as low back pain, neuropathic pain, fibromyalgia, or complex regional pain syndrome, who had been referred to tertiary pain care by their physicians after inadequate treatment response in primary care. Patients having active cancer were not recruited to the study. KROKIETA collected a broad range of data, including sociodemographic, health, psychological, lifestyle, and biochemical variables.^35,55^ Patients were first screened for their subjective level of sleep difficulties by the sleep item in the 15D HRQoL questionnaire^46^ (Fig. 1; see Measures). Those reporting normal sleeping were assigned to the “Sleeping normally” group. Those reporting at least marked sleep difficulties were further examined for sleep problems by items in the Basic Nordic Sleep Questionnaire (BNSQ)^40^: those with recurring problems (see Measures) were assigned to the “Sleep problems” group.

The established groups were compared for demographics, pain variables, pain diagnoses, pain-related anxiety, the use of sleep and pain medications, childhood adversities, self-reported diseases, and the incidence of sleep disorders. To investigate further the role of pain-related anxiety components in relation to sleep problems, these were assessed for their importance in more disturbing sleep problems in the whole study cohort. This was conducted in a series of regression models, first with individual pain-related anxiety components as predictors, after which a full model was constructed. This also included basic pain variables, based on their known association with sleep problems: pain intensity, number of pain areas, and pain duration.^20,24,32^

The study protocol (29/13/03/00/12) was approved by the coordinating ethics committee of the Helsinki and Uusimaa Hospital District. The STROBE guidelines for reporting observational studies (www.equator-network.org) were followed in this study.

### 2.2. Data collection

Patients were enrolled in the study between September 2013 and November 2016, receiving an invitation to participate in the same mailing in which they received their first appointment at the pain clinic. The mailing included the regular preappointment questionnaires on pain, health, sleep, and lifestyle factors, which patients completed at home. During the first visit to the clinic, patients who agreed to participate in the study completed further questionnaires on psychological factors, nutrition, substance use, and quality of life. All patients in the study provided written informed consent. Exclusion criteria were active cancer and inability to answer questionnaires in Finnish.

### 2.3. Measures

Recurring sleep problems were assessed with the following criteria. Subjective sleep difficulties were queried with the sleep item from 15D HRQoL questionnaire.^46^ 15D is a generic, standardized questionnaire, comprising 15 items assessing different dimensions of health (eg, mobility, sleeping, mental function etc), validated with chronic pain patients.^55^ The sleep item asks respondents to indicate whether they are sleeping normally or having mild, marked, great, or extreme sleep difficulties. Patients indicating normal sleeping were assigned as “Sleeping normally.” Those indicating at least marked difficulties were further assessed for recurring sleep problems with the BNSQ.^40^ This was developed as a standardized questionnaire to assess sleep disorders, such as difficulties initiating or maintaining sleep, OSA, or RLS. It queries different symptoms over the past 3 months on a scale of 1 (never or less than once per month); 2 (less than once per week); 3 (1-2 nights per week); 4 (3-5 nights per week); and 5 (every night or almost every night). Recurring sleep problems were assessed with 3 core questions^49,59^: difficulties falling asleep; repeated nocturnal awakenings; and unrefreshing sleep. Patients were assigned to the “Sleep problems” group if they reported having experienced at least one of the following problems:

(1) difficulties falling asleep at least 3 times per week,
(2) nocturnal awakenings at least 3 times per night, on at least 3 nights per week,
(3) feeling extremely tired after waking up in the morning at least 3 times per week, and in addition, having had daytime tiredness at least 3 times per week. The criteria were set to select those with recurring sleep problems that would also impact on daytime functioning.

Demographics were collected from the preappointment questionnaire. Pain intensity was assessed with the Brief Pain Inventory (BPI),^15^ which measures pain intensity with 4 items (worst, least, on average, and currently), from which the sum score was calculated. The items in the BPI are answered on a numerical rating scale ranging from 0 to 10. The psychometric properties of the BPI are well established.^14,26^ The number of pain sites was calculated from a figure of the human body in the preappointment questionnaire, where the patient had marked all areas with pain. The figure was divided into 11 subareas (head, face, front of neck, back of neck, shoulders, chest, upper limbs, stomach, upper back, lower back, and lower limbs). The duration of pain was queried with a six-interval scale (<1 month, 1-3 months, 3-6 months, 6-12 months, 1-2 years, and > 2 years). Pain diagnoses were assessed and recorded by the physician after patient examination in the first visit to the pain clinic.

Pain-related anxiety was assessed with the Pain Anxiety Symptoms Scale-20 (PASS-20).^34^ This measures 4 anxiety components in relation to pain: cognitive anxiety (example item: “I worry when I am in pain”); escape/avoidance behaviour (“I avoid important activities when I hurt”); fear of pain (“When I feel pain I am afraid that something terrible will happen”); and physiological anxiety (“Pain seems to cause my heart to pound or race”). The measure comprises 20 items, which the patient answers on a 6-interval scale (from “never” to “always”). This yields scores between 0 and 25 on all scales. The factor structure and internal consistencies (Cronbach α = 0.75-0.86) of the scales have been confirmed.^34^

Childhood adversities were queried as in the Health 2000 epidemiological survey^41^ with 11 items, asking whether any of the following were present before the age of 16 yeras: financial difficulties in the family, parental unemployment, parental serious illness, paternal alcohol abuse, maternal alcohol abuse, paternal serious psychiatric condition, maternal serious psychiatric condition, serious conflicts within family, parental divorce, own serious illness, and bullying at school.

Frequency of sleep medication use over the past 3 months was queried with an item in the BNSQ on a scale of 1 (never or less than once per month); 2 (less than once per week); 3 (on 1-2 nights per week); 4 (on 3-5 nights per week); and 5 (every night or almost every night). The use of different sleep medications was extracted from the list of medications used that the patient provided in the preappointment questionnaire. The current regular use of pain medications was recorded in detail by a physician during examinations in the pain clinic. For the analysis, medications were grouped as follows: paracetamol (acetaminophen); nonsteroidal anti-inflammatory drugs; amitriptyline/nortriptyline; venlafaxine/duloxetine; carbamazepine/oxcarbazepine/lamotrigine; gabapentin/pregabalin; codeine/tramadol; buprenorphine; and oxycodone/morphine/hydromorphone/fentanyl.

Self-reported diseases were recorded from the preappointment questionnaire, where patients were asked whether they had been diagnosed or treated by a physician within the previous 12 months for the following: cardiovascular diseases (hypertension, heart failure, and angina pectoris), diabetes mellitus, lung diseases (pulmonary asthma and chronic obstructive pulmonary disease), musculoskeletal diseases (rheumatoid arthritis [RA], another articular disease, and low back problem), or psychiatric diseases (depression or some other psychiatric condition).

The sleep disorders RLS and OSA were assessed by BNSQ items.^40^ Patients were asked about RLS, described as comprising the following features^1^: (1) the urge to move the legs when sitting or lying down; (2) accompanying dysaesthesia; (3) symptoms relieved by movement; and (4) symptoms becoming worse during the evening/night. Restless legs syndrome was diagnosed if the symptoms had been present on 3 or more nights per week during the past 3 months. Obstructive sleep apnoea was assessed as in previous studies^38^: self-reported OSA was diagnosed if the patient reported frequent snoring (on 3 or more nights per week) and, in addition, reported (1) loud and irregular snoring, with occasional respiratory pauses and/or stertorous breathing, or (2) respiratory pauses, on one or 2 nights per week.

### 2.4. Statistical analysis

Missing data were screened first. Where patients had more than 2 missing answers on the 4 BNSQ items used for sleep problem classification, their data were considered missing. For those omitting one or 2 answers (9 patients), the missing data were imputed with the median of BNSQ items that were available. Group differences were analysed with chi-squared, *t*, and Mann–Whitney *U*-tests, as appropriate. Values of *P* < 0.05 were considered significant in these and all following analyses.

To investigate the role of pain-related anxiety components in relation to sleep problems, the components were compared for importance in modelling more disturbing sleep problems in a series of cumulative odds ordinal regression analyses with proportional odds in the whole study cohort. The assumption of proportional odds was assessed by a full likelihood ratio test comparing the fitted model to a model with varying location parameters. The dependent variable included 3 groups: (1) sleeping normally; (2) mild or infrequent sleep problems (intermediate group between the 2 extremes in the whole cohort); and (3) sleep problems. Components were first entered individually as the only predictor variable to the model and then compared for importance with the resulting model fit indices (Nagelkerke pseudo *R*^2^), with the highest value indicating the greatest importance.^29^ A full model was then constructed with the predictors “pain intensity,” “number of pain areas,” and “pain duration” (included on theoretical grounds for their established association with sleep problems), and the “pain-related anxiety” variable with the highest fit index value when modelling individually. Then, to further evaluate variable importance, the chosen pain-related anxiety variable was inspected as to whether it would remain statistically significant when the 3 remaining pain-related anxiety variables were, one at a time, added to the model. SPSS 25.0 software program package (IBM Corp. Released 2017. IBM SPSS Statistics for Windows. Armonk, NY: IBM Corp) was used for statistical analyses.

## 3. Results

### 3.1. Demographics and pain

The whole cohort comprised 473 patients (69.9% female; age range 18-81 years; mean ± SD: 47.0 ± 13.8 years). Data were available to assign as sleeping normally or having sleep problems for 458 patients in the cohort. Of these, 61 patients (13.3% of the cohort) reported sleeping normally, whereas 199 (43.4%) reported at least “marked” sleeping difficulties and fulfilled the criteria for recurring sleep problems. The remaining 198 patients were between these 2 groups, reporting only “mild” or infrequent problems with sleep (present only 1-2 times per week or less).

Those sleeping normally and those having recurring sleep problems did not differ in age, sex, or years of education (Table 1). Patients with sleep problems were less often working or studying (38.1% vs 59.0%, *P* = 0.004) and more often living alone (28.1% vs 14.8%, *P* = 0.04) than those sleeping normally.

**Table 1:** Demographic and pain variables in chronic pain patients sleeping normally or having sleep problems.

	Sleeping normally	Sleep problems	*P*
n	61	199	
Age: mean (SD)	46.7 (14.7)	46.5 (12.7)	0.89
Female: n (%)	42 (68.9)	134 (67.3)	0.83
Education years: mean (SD)	13.7 (3.7)	13.7 (3.8)	0.98
Living alone: n (%)	9 (14.8)	56 (28.1)	**0.04**
Currently working or studying: n (%)	36 (59.0)	75 (38.1)	**0.004**
Pain intensity: mean (SD)	4.2 (2.2)	6.3 (1.5)	**<0.001**
Number of pain sites: median (IQR)	2.0 (4)	4.0 (5)	**<0.001**
Pain duration > 2 y: n (%)	36 (61.0)	153 (78.1)	**0.01**
PASS-20: mean (SD)			
Cognitive anxiety (0-25)	10.8 (6.9)	15.4 (5.1)	**<0.001**
Escape/avoidance (0-25)	9.7 (6.5)	13.4 (4.9)	**<0.001**
Fear of pain (0-25)	6.7 (5.4)	10.5 (5.7)	**<0.001**
Physiological anxiety (0-25)	4.7 (5.0)	9.7 (5.3)	**<0.001**
n*	58	196	
Pain diagnoses: n (%)			
Neuropathic pain	15 (25.9)	57 (29.1)	0.63
Back pain	6 (10.3)	52 (26.5)	**0.01**
Other musculoskeletal pain	9 (15.5)	49 (25.0)	0.13
Facial pain	37 (63.8)	39 (19.9)	**<0.001**
Headache	4 (6.9)	13 (6.6)	1.000
Fibromyalgia	2 (3.4)	22 (11.2)	0.08
Abdominal pain	0 (0.0)	6 (3.1)	0.34
CRPS	1 (1.7)	11 (5.6)	0.31
Phantom limb pain	0 (0.0)	1 (0.5)	1.000
Chronic pain syndrome	2 (3.4)	5 (2.6)	0.66

* Differences in n with respect to original categorization are due to missing data. Bold indicates *P* values <0.05.

CRPS, complex regional pain syndrome; IQR, interquartile range; PASS-20, Pain Anxiety Symptoms Scale-20.

Patients having sleep problems had 2.08 points higher mean pain intensity (95% CI: 1.48-2.67; *P* < 0.001), greater median number of pain sites (4.0 vs 2.0, *P* < 0.001), and pain duration > 2 years (78.1% vs 61.0%; *P* = 0.01) than patients sleeping normally. Patients having sleep problems had significantly more pain-related anxiety than those sleeping normally in all assessed components. Mean cognitive anxiety was 4.7 points (95% CI: 2.7-6.6), escape/avoidance behaviour 3.7 points (95% CI: 1.8-5.5), fear of pain 3.8 points (95% CI: 2.1-5.5), and physiological anxiety 5.0 points (95% CI: 3.5-6.6) (all *P* < 0.001) higher in those with sleep problems than in those who slept normally. Patients having sleep problems more often had a back pain diagnosis (26.5% vs 10.3%; *P* = 0.01) and less often a facial pain diagnosis (19.9% vs 63.8%; *P* < 0.001) than did normally sleeping patients. Because having facial pain or back pain diagnoses appeared as significant factors in relation to sleep problems, an additional analysis was conducted to clarify the differences between these groups, showing that back pain patients had higher pain intensity and more comorbid conditions (Supplemental Table 1, available at http://links.lww.com/PAIN/B507).

### 3.2. Childhood adversities

Those having sleep problems and those sleeping normally did not differ in median numbers of childhood adversities experienced or incidences of individual adversities (Supplemental Table 2, available at http://links.lww.com/PAIN/B507). The only difference between the groups appeared in the cumulative number of adversities, where 5 or more was 3 times as common in those with sleep problems (15.3%) than those sleeping normally (5.1%) (*P* = 0.04). In the whole cohort, the most common adversities experienced were parents' divorce (29.0%), bullying at school (28.8%), and own serious illness (12.7%). 75.3% of all patients in the cohort reported at least one childhood adversity.

### 3.3. Medication use

27.8% of the patients with sleep problems used prescribed sleep medications regularly (on 3 or more nights per week), whereas only 3.3% of those sleeping normally reported this (*P* < 0.001) (Table 2). Zolpidem and melatonin were the most commonly used sleep-affecting medicines among patients with sleep problems (both used by 12.6% of patients).

**Table 2:** Use of sleep and pain medications in chronic pain patients sleeping normally or with sleep problems.

n*	Sleeping normally	Sleep problems	*P*
	61	198	
Using prescribed sleep medication regularly (3 or more nights per week): n (%)	2 (3.3)	55 (27.8)	**<0.001**
Reported use of: n (%)			
Benzodiazepine derivatives			
Temazepam	0 (0.0)	11 (5.5)	0.06
Diazepam	0 (0.0)	9 (4.5)	0.12
Zolpidem	0 (0.0)	25 (12.6)	**0.004**
Melatonin	1 (1.6)	25 (12.6)	**0.01**
Quetiapine	0 (0.0)	11 (5.5)	0.07
Mirtazapine	1 (1.6)	11 (5.5)	0.31
Pain medications in regular use: n (%)			
Paracetamol (acetaminophen)	5 (8.2)	34 (17.2)	0.09
NSAIDs	3 (4.9)	48 (24.2)	**0.001**
Amitriptyline/nortriptyline	5 (8.2)	39 (19.7)	**0.04**
Venlafaxine/duloxetine	1 (1.6)	22 (11.1)	**0.02**
Carbamazepine/oxcarbazepine/lamotrigine	8 (13.1)	7 (3.5)	**0.01**
Gabapentin/pregabalin	7 (11.5)	51 (25.8)	**0.02**
Codeine/tramadol	2 (3.3)	52 (26.3)	**<0.001**
Buprenorphine	4 (6.6)	20 (10.1)	0.40
Oxycodone/morphine/hydromorphone/fentanyl	0 (0.0)	6 (3.0)	0.34

* Differences in n with respect to original categorization are due to missing data. Bold indicates *P* values <0.05.

NSAIDs, nonsteroidal anti-inflammatory drugs.

Pain medications more often regularly used by those with sleep problems than by the normally sleeping group included codeine-combinations/tramadol (26.3% vs 3.3%; *P* < 0.001), gabapentin/pregabalin (25.8% vs 11.5%; *P* = 0.02), NSAIDs (24.2% vs 4.9%; *P* = 0.001), amitriptyline/nortriptyline (19.7% vs 8.2%; *P* = 0.04), and venlafaxine/duloxetine (11.1% vs 1.6%; *P* = 0.02). Those with sleep problems were using less often carbamazepine/oxcarbazepine/lamotrigine than those sleeping normally (3.5% vs 13.1%; *P* = 0.01).

### 3.4. Self-reported diseases and sleep disorders

Those having sleep problems had a higher incidence of angina pectoris (6.5% vs 0.0%; *P* = 0.04), asthma (19.6% vs 1.7%; *P* = 0.001), non-RA joint disease (32.3% vs 18.3%; *P* = 0.04), low back problems (55.1% vs 23.3%; *P* < 0.001), and depression (31.6% vs 5.0%; *P* < 0.001), than those who slept normally (Table 3). Those sleeping normally reported more RA than those with sleep problems (11.7% vs 2.5%; *P* = 0.01). Regarding depressive symptoms, the mean BDI-II score was significantly higher in those with sleep problems than those sleeping normally (18.4 vs 5.7; *P* < 0.001). 68.6% of the patients with sleep problems who reported comorbid depression during the past 12 months had at least mild symptoms of depression (BDI-II score > 13), whereas 38.2% had moderate to severe symptoms (score > 19). Restless legs symptoms were significantly more frequent among those with sleep problems than in those sleeping normally (33.2% vs 11.7%; *P* = 0.001). Differences between the groups in numbers of patients with self-reported OSA were not statistically significant.

**Table 3:** Self-reported diseases and sleep disorders in chronic pain patients sleeping normally or with sleep problems.

n*	Sleeping normally	Sleep problems	*P*
	60	199	
Self-reported diseases: n (%)			
Hypertension	14 (23.3)	63 (31.7)	0.22
Heart failure	2 (3.3)	4 (2.0)	0.63
Angina pectoris	0 (0.0)	13 (6.5)	**0.04**
Diabetes	6 (10.0)	22 (11.1)	0.81
Asthma	1 (1.7)	39 (19.6)	**0.001**
COPD	1 (1.7)	7 (3.5)	0.69
RA	7 (11.7)	5 (2.5)	**0.01**
Other joint disease	11 (18.3)	64 (32.3)	**0.04**
Low back problems	14 (23.3)	109 (55.1)	**<0.001**
Depression	3 (5.0)	62 (31.6)	**<0.001**
Psychiatric disorder other than depression	1 (1.7)	18 (9.0)	0.09
Sleep disorders			
RLS symptoms (3 or more nights per week): n (%)	7 (11.7)	66 (33.2)	**0.001**
Self-reported OSA: n (%)	4 (6.7)	30 (15.1)	0.12

* Differences in n with respect to original categorization are due to missing data. Bold indicates *P* values <0.05.

COPD, chronic obstructive pulmonary disease; OSA, obstructive sleep apnoea; RA, rheumatoid arthritis; RLS, restless legs syndrome.

### 3.5. Regression analyses with pain-related anxiety

When pain-related anxiety components were investigated individually for their importance in more disturbing sleep problems with a series of ordinal logistic regressions (including the entire cohort, ie, the dependent variable included patients sleeping normally, those with mild/infrequent problems, and those in the sleep problems group), physiological anxiety showed the highest model fit value of 0.137 (Nagelkerke pseudo *R*^2^), followed by cognitive anxiety (0.095), fear of pain (0.080), and escape/avoidance (0.058). Pain intensity alone resulted in a model with fit value of 0.192.

A full model, with pain intensity, number of pain areas, pain duration > 2 years, and physiological anxiety as predictors, statistically significantly predicted the dependent variable over and above the intercept-only model χ^2^(4) = 111.278, *P* = < 0.001; Nagelkerke pseudo *R*^2^ = 0.265 (Table 4). Pain lasting over 2 years did not remain significant in the model. When cognitive anxiety, fear of pain, or escape/avoidance was added to the model beside physiological anxiety, physiological anxiety remained significant, whereas the added variable was nonsignificant. Therefore, as physiological anxiety provided the best fit on its own and remained as the only statistically significant pain-related anxiety component in the model when the other components were added, it appeared in the analyses as the statistically most important factor in more disturbing sleep problems.

**Table 4:** Ordinal logistic regression final full model in the whole cohort.

Full model: grouping of the sleep problems (sleeping normally, mild/infrequent problems, sleep problems group) as the dependent variable	B	SE (B)	OR	95% CI	*P*
Threshold					
Sleeping normally	1.272	0.366	3.568	1.740-7.315	**<0.001**
Mild/infrequent problems	3.924	0.414	50.599	22.481-113.883	**<0.001**
Pain intensity	0.433	0.062	1.541	1.365-1.740	**<0.001**
Number of pain sites	0.079	0.040	1.082	1.001-1.170	**0.046**
Pain duration > 2 y	0.391	0.218	1.478	0.965-2.264	0.073
PASS-20 physiological anxiety	0.077	0.020	1.080	1.039-1.124	**<0.001**

Nagelkerke pseudo R^2^ = 0.265. Bold indicates *P* values <0.05.

CI, confidence interval; OR, odds ratio; PASS-20, Pain Anxiety Symptoms Scale-20.

## 4. Discussion

In this study investigating patients entering tertiary pain care, 43.4% were classified as having recurring sleep problems. The main findings of the study indicate that the physiological component of pain-related anxiety appears as a significant factor in the relation between pain and sleep; those patients with sleep problems report a number of co-occurring health conditions (depression, angina pectoris, asthma, low back problem, and non-RA joint disease) more often than those sleeping normally; restless legs symptoms constitute a significant sleep disorder; and patients with sleep problems report more regular use of sleep medications and also use more pain medications.

### 4.1. Physiological reactions to pain seem important

Physiological anxiety, a subscale of PASS, seemed to be the most important component of pain-related anxiety associated with more disturbing sleep problems. Previous research has associated pain catastrophizing^11,12,56^ with sleep problems, with component analyses indicating rumination as the key psychological process. Physiological reactions to pain may not have received enough attention in previous research, as the often-used Pain Catastrophizing Scale^48^ does not cover bodily reactions. The results here clearly indicate that, along with pain intensity and cognitive processes, physiological anxiety reactions to pain should be investigated in relation to pain and sleep.

The physiological anxiety component reflects a patient's experience when it is difficult to calm down, the body is trembling, and the heart racing, while in pain.^34^ It is easy to imagine that, if one has pain at bedtime, these reactions will make it difficult to fall asleep. But heightened physiological arousal may affect sleep problems in other ways. Arousal seems to lead to overestimation of sleep onset latency and underestimation of total sleep time,^50^ and these cognitive misperceptions may add to anxiety and behavioural patterns that cause sleep problems on their own.^21^ Regarding treatment, cognitive–behavioural interventions and relaxation skills training may provide help in addressing physiological anxiety reactions.

### 4.2. Self-reported diseases and restless legs

In a multicountry study, Koyanagi et al.^28^ showed a linear dose-dependent correlation between the number of chronic conditions (angina pectoris, arthritis, asthma, chronic lung disease, depression, diabetes, hypertension, obesity, and stroke) and sleep problems. When observed separately, angina (OR 1.75-2.78), arthritis (OR 1.39-2.46), and depression (OR 1.75-5.12) were the conditions most often associated with sleep problems. Here as well, as expected, those pain patients with sleep problems more frequently reported health conditions (angina pectoris, asthma, and depression) than those sleeping normally. In particular, the difference in asthma reports in the groups (1.7% vs 19.6% in the sleep problems group) is noteworthy. Luyster et al.^31^ showed that insomnia was common (37%) among those with asthma and it was also associated with worse asthma control. Asthma can lead to sleep disturbance via several physiological mechanisms, such as increase in bronchial hyperresponsiveness.^25^ Furthermore, asthma and OSA are mutually associated. However, Brumpton et al.^10^ showed that insomnia tripled the risk of developing asthma over an 11-year follow-up period.

Chronic pain, depression, and sleep problems are already known to have bidirectional relationships^18,20,42^; so, it was therefore not unexpected that so many (31.6%) among those with sleep problems suffered physician-diagnosed depression in the past year. However, this finding underlines the fact that, along with pain and anxiety, depression is frequently involved in the sleep problems of pain patients. It seems that sleep problems are an independent risk factor for the development of depression,^18^ possibly through, for example, increase in inflammatory substances, decrease in monoamines (such as serotonin, noradrenalin, and dopamine), or alterations in circadian rhythm processes. These same factors are also of interest for chronic pain development.^4,6,45^

Back pain, joint diseases, and RA have previously been shown to be associated with increased sleep problems,^2,36,52^ and here too, back pain and joint diseases were more common in those with sleep problems. However, surprisingly, arthritis was more common among those sleeping normally than those with sleep problems. In the sample, arthritis was rarely the main cause for pain, but a co-occurring disease, the symptoms of which are today usually well controlled pharmacologically.

The prevalence of RLS in those having sleep problems, 33.2%, was nearly three-fold of that in those sleeping normally, and markedly higher than estimates of 5% to 10% in the general population.^17^ Factors suggested to cause or to be associated with RLS include genetic predisposition, brain iron deficiency, and alterations in dopaminergic metabolism or circuitry. Dopaminergic mechanisms may link RLS and widespread pain.^47^ Also, sleep problems in RLS are associated with anxiety and depression,^8,58^ and therefore, as in pain, the latter may be contributing factors in sleep problems. The prevalence of OSA in those with sleep problems was twice that of those sleeping normally, but the difference was not statistically significant.

### 4.3. Use of medications

Not surprisingly, pain and sleep medication utilization was significantly higher in the sleep problems group. However, some of the medications used can be considered problematic for sleep, such as the most commonly used benzodiazepine derivatives. Their high use may be partly attributable to the underlying problem of anxiety in these patients. Benzodiazepines affect sleep structure negatively, while one reason for continuing their use includes dependence.^5^ Also, analgesics have both beneficial and detrimental effects on sleep, although research is limited.^7^ If medications affect sleep, this may complicate efforts to alleviate the comorbidity of pain and sleep problems. Therefore, nonpharmacological methods to improve sleep would be preferable.^44^

### 4.4. Pain aetiology and childhood adversities

Facial pain patients have fewer sleep problems than do back pain patients, but the reasons are not clear.^39^ This difference was also found in our study, with additional analysis revealing higher pain intensity and longer pain duration on average, and more RLS, asthma, and psychological distress in those with back pain than in those with facial pain. Thus, back pain patients seem to have more complicating factors influencing sleep than do facial pain patients, but the reason for this remains an interesting question for further studies.

Childhood adversities are known to be associated with more somatic and mental health problems^23^ but, in our study, differences between those having sleep problems and those sleeping normally appeared only when the cumulative number of adversities amounted to 5 or more. Comparing the whole cohort data with an epidemiological study (n = 4076) using the same questionnaire,^41^ it should be noted that the incidence of maternal alcohol abuse was nearly five-fold, parents' divorce rate three-fold, and paternal psychiatric problems two-fold higher with the pain patients than in the general population. Because childhood adversities alone may predispose to the development of pain problems,^27,30,37^ this may mask their effects on sleep problems. The key factor seems to be the accumulation of adversities.^13,19,57^

### 4.5. Problem cycles as treatment targets

**Figure 2.**
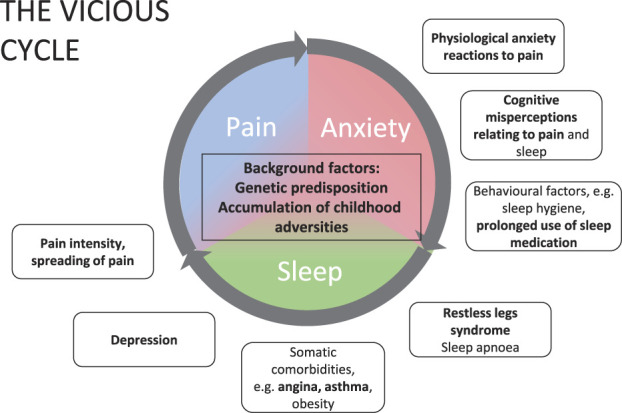
A proposed problem cycle in comorbid pain and sleep problem. Reciprocal relationships between pain, anxiety, and sleep make self-reinforcing problem cycles plausible. An example of the process might depict acute pain, which in individuals with vulnerabilities (eg, genetic or enhanced stress reactivity) evokes heightened anxiety, leading to problems in sleep initiation and continuation. Accumulating anxiety and reduced sleep may lead to more intense and widespread pain, and vice versa. Outside of the core cycle are listed subprocesses of the factors in the cycle (eg, physiological anxiety reactions) and factors with interrelationships to the cycle (eg, RLS). These may serve as treatment targets to interrupt the cycle. Factors in bold appeared in this study to have significant associations with recurring sleep problems. RLS, restless legs syndrome.

There is still little knowledge of the mechanisms of development of comorbid pain and sleep problems over time. Several factors may initiate the process, which is then advanced by reciprocal effects.^51^ It seems likely that with reciprocal relationships between many factors, there is a risk of self-reinforcing cycles, which may be involved in the development or maintenance of problems. Figure 2 depicts one possible “vicious” cycle, involving pain, anxiety, and sleep problems. Only modest improvements in sleep occur when treatment is targeted solely on pain. Targeting subprocesses (such as physiological anxiety reactions) or possible interacting factors (such as RLS) may provide ways to break these cycles and achieve better outcomes.

### 4.6. Strengths and limitations

Research is still clarifying the range of factors involved in co-occurring pain and sleep problems. With a large selection of included variables, this study contributes to that goal. A cohort with mixed pain aetiologies decreases the likelihood that findings are limited to specific pain conditions. As a limitation, it should be noted that self-report measures carry the possibility of bias, such as when anxiety may lead to overestimation of sleep difficulties.^50^ Sleep disorder assignments were based on self-report but followed previous research in their operationalizations. Patients in the cohort represent those in specialized care, therefore having more severe pain: generalization to all chronic pain patients may be limited. Finally, the cross-sectional study setting does not allow any conclusions about causal relationships between the factors.

## 5. Conclusions

A high proportion of pain patients entering tertiary care suffer from recurring sleep problems. The results of this study reinforce the belief that sleep problems in pain patients are likely to be highly multifactorial. Alongside previously established factors, such as pain intensity and depression, this study draws attention to physiological anxiety reactions, co-occurring diseases (such as angina pectoris and asthma), restless legs symptoms, and the use of sleep and pain medications. The careful assessment of all these factors and planning treatment accordingly is the key for addressing sleep problems in pain patients. Acknowledging these factors as early as possible in primary care may even halt the development of sleep problems for some patients.

## Conflict of interest statement

E. Kalso reports personal fees for advisory board work from Pfizer, outside the submitted work. The remaining authors have no conflicts of interest to declare.

## Appendix A. Supplemental digital content

Supplemental digital content associated with this article can be found online at http://links.lww.com/PAIN/B507.
